# CpG-oligodeoxynucleotides challenged macrophages ameliorate acetaminophen induced liver injury by activating TLR9/IRG1/itaconate metabolic pathway

**DOI:** 10.1186/s10020-025-01324-0

**Published:** 2025-08-25

**Authors:** Yibai Qu, Zehui Jiang, Zhixia Chen, Sidan Luo, Bingyao Xie, Xubo Wu, Gang Yuan, Kan Wu, Li Chen, Tian Tian, Shan Li, Haihua Luo, Quan Li, Ding Yuan, Yan Zhang, Yanxia Gao, Jun Zhou, Zhengzheng Yan, Yong Jiang

**Affiliations:** 1 Department of Anesthesiology, The Third Affiliated Hospital of Southern Medical University, Guangzhou, 510630 China; 2 Department of Anesthesiology, The Tenth Affiliated Hospital (Dongguan People’s Hospital), Southern Medical University, Dongguan, Guangdong 523059 China; 3 Dongguan Key Laboratory of Anesthesia and Organ Protection, Dongguan, 523059 Guangdong China; 4 Guangdong Provincial Key Laboratory of Proteomics, School of Basic Medical Sciences, Southern Medical University, Guangzhou, 510515 China; 5 Department of Anesthesiology, National Cancer Center/National Clinical Research Center for Cancer/Cancer Hospital and Shenzhen Hospital, Chinese Academy of Medical Sciences and Peking Union Medical College, Shenzhen, 518116 China; 6 Department of Hepatobiliary and Pancreatic Surgery, Minhang Hospital, Fudan University, Shanghai, 201199 China; 7 State Key Laboratory of Metabolic Dysregulation & Prevention and Treatment of Esophageal Cancer, Henan Key Laboratory of Critical Care Medicine, Henan International Joint Laboratory of Infection and Immunology, Department of Emergency Medicine, The First Affiliated Hospital, Zhengzhou University, Zhengzhou, 450001 China; 8 Institute of Infection and Immunity, Henan Academy of Innovations in Medical Science, Zhengzhou, China; 9 Department of Emergency Medicine, Henan Provincial Medical Key Laboratory of Toxicological Diseases, The First Affiliated Hospital of Zhengzhou University, Zhengzhou, 450001 China

**Keywords:** Acetaminophen, IRG1, Itaconate, Macrophage apoptosis, Liver injury, Mitochondrial dysfunction

## Abstract

**Background:**

Acetaminophen, or *N*-acetyl-para-aminophenol (APAP), causes severe liver damage and acute liver failure when overdosed. Oligodeoxynucleotides containing CpG motifs (CpG ODN) can regulate the function of macrophages, which play an important role in drug-induced liver injury. It is unclear whether CpG ODN-treated macrophages play an immune regulation role in APAP-induced liver injury. In the present study, we aim to explore the role of CpG ODN-activated macrophages in APAP-induced liver injury and the underlying mechanism in protecting against the cytotoxicity of APAP.

**Methods:**

In vivo, C57BL/6 mice were treated with APAP (300 mg/Kg) or/and CpG ODN (ODN 1826, 1.65 mg/Kg) by intraperitoneal injection, then survival rate, histopathological evaluation, and inflammatory factors were observed to ascertain the protective effect of CpG ODN. Then, CpG ODN-treated macrophages were reinfused into the animal model to determine the effector cells. In vitro, RNA sequencing and untargeted metabolomics detection were performed to illustrate the underlying mechanism. Last, *Acod1* siRNA interference was used to clarify the role of IRG1 in resistance to APAP cytotoxicity by ROS and apoptosis indicator detections.

**Results:**

We found that CpG ODN showed a protective effect against APAP cytotoxicity by stimulating macrophages rather than hepatic parenchymal cells. In particular, reinfusion of CpG ODN-treated macrophages to mice can alleviate APAP-induced liver injury. Transcriptome and metabolome analysis revealed that the expression of aconitate decarboxylase 1 (*Acod1*; also known as immune responsive gene 1, IRG1) and the metabolite itaconate generated by IRG1 catalysis increased after CpG ODN stimulation. In addition, we found that the mechanism of this protective effect is ascribed to the increased expression of *Acod1* and the antioxidative function of itaconate by the activation of the TLR9/NF-κB signaling pathway.

**Conclusion:**

CpG ODN alleviated liver injury induced by APAP through the activation of the TLR9/NF-κB signaling pathway in macrophages, upregulating the expression of IRG1 protein, promoting the production of endogenous metabolite itaconate, and inhibiting macrophage apoptosis which was regulated by upregulating the expression of Nrf2 to inhibit ROS production. This study sheds new light on CpG ODN as a therapeutic strategy in resistance to APAP-induced liver injury.

**Supplementary Information:**

The online version contains supplementary material available at 10.1186/s10020-025-01324-0.

## Introduction

Acetaminophen, also known as *N*-acetyl-para-aminophenol (APAP) or paracetamol, is one of the most commonly used drugs owing to its analgesic and antipyretic effects (Yoon et al. 2016). It is safe and effective at recommended doses; however, overdose may lead to significant hepatotoxicity and even acute liver failure (ALF) (Bunchorntavakul and Reddy 2018). According to epidemiologic studies, APAP hepatotoxicity is the most frequent cause of ALF in many countries (Bunchorntavakul and Reddy 2018; Ramachandran and Jaeschke 2019; Gulmez et al. 2015).

N-acetyl cysteine (NAC) is recommended as an effective clinical antidote against APAP poisoning, especially when used in the early phase (Du et al. 2016). However, the disadvantages of using NAC for the clinical treatment of APAP cytotoxicity also need to be considered (Yan et al. 2018).Therefore, new interventions and strategies must be developed to improve the therapeutic effectiveness of APAP-induced liver injury.

Early mechanistic insight into the pathophysiology of APAP hepatotoxicity was always based on intracellular events in the hepatocytes (Ramachandran and Jaeschke 2019; Nguyen et al. 2021). Generally, APAP-induced oxidative stress and mitochondrial dysfunction are considered the predominant intracellular events in the pathogenesis of drug-induced acute liver injury. However, many other cellular processes including sterile inflammation, endoplasmic reticulum stress, microcirculatory disturbance, autophagy, and liver regeneration may participate in the pathogenesis of APAP-induced acute liver injury (Jaeschke and Ramachandran 2020; Pu et al. 2023; Ito et al. 2004; Zhao et al. 2021; Bhushan and Apte 2019). Nuclear factor erythroid 2-related factor 2 (Nrf2) orchestrates the expression of a broad array of antioxidant enzymes that mitigate oxidative damage through detoxification and clearance mechanisms, with its therapeutic and pathophysiological roles being widely explored in various disease models (Ngo and Duennwald 2022). Functioning as a transcription factor, Nrf2 directly binds to the heme oxygenase-1 (HO-1) promoter to modulate HO-1 gene expression. The Nrf2/HO-1 signaling axis serves as a critical defense mechanism against diverse pathological stressors, including oxidative damage, inflammatory cascades, and apoptotic cell death. This evolutionarily conserved pathway coordinates systemic adaptive responses by upregulating cytoprotective genes, thereby conferring resilience to both endogenous metabolic disruptions (e.g., mitochondrial dysfunction) and exogenous toxicants (e.g., environmental pollutants). Its cardinal functions encompass the neutralization of reactive oxygen species (ROS), suppression of pro-inflammatory mediators, and inhibition of caspase-dependent apoptosis, making it indispensable for maintaining cellular homeostasis across multiple organ systems. Consequently, targeting the Nrf2/HO-1 signaling pathway would be a promising strategy in mitigating APAP cytotoxicity (Zhang 2025). APAP cytotoxicity is known to directly damage the parenchymal cells of the hepatocytes. There is an increasing awareness that non-parenchymal hepatic cells and infiltrating inflammatory cells are involved in the pathogenesis of APAP-induced liver injury (Li et al. 2023; Zeng et al. 2024). New insights into APAP-induced liver injury have focused on the role of infiltrating immune cells in the pathogenesis. Studies have shown that innate immune cells within the liver, such as hepatic macrophages, natural killer (NK) cells, NKT cells, and polymorph nuclear leukocytes (PMNs), are activated by impaired hepatocytes (Liu et al. 2004; Guo et al. 2021). These immune cells are involved in the progression of APAP-induced liver injury. Recent studies on the pathogenesis of APAP-induced liver injury also revealed that cell death in hepatocytes and immune cells residing in the liver or circulation determines the severity and outcome of the disease (Maes et al. 2016).

Macrophages are the most important innate immune cells that sense and respond to invading pathogens and form the first line of the human immune defense system. These cells play a pivotal role in increasing the levels of inflammatory cytokines and participating in tissue repair after liver injury. However, the role of macrophages in APAP-induced liver injury pathogenesis remains controversial. It is known that hepatic macrophages conduce to APAP-induced hepatotoxicity by generating pro-inflammatory cytokines and mediators, as well as tumor necrosis factor α (TNF-α), interleukin-1β (IL-1β), and nitric oxide (NO). While, hepatic macrophages also exert hepatoprotection by generating cytokines and mediators such as IL-10, IL-6, and IL-18 binding proteins, which weaken the inflammatory response and promote liver repair and regeneration (Ghaffari et al. 2011). There is evidence to support that macrophages play a key role in tissue repair following APAP-induced liver injury (Osawa et al. 2010; Owumi et al. 2014; Abshagen et al. 2007; Izumi et al. 2018).

Synthetic oligodeoxynucleotides containing an unmethylated CpG motifs (CpG ODN), which specifically trigger the toll like receptor (TLR) 9-mediated innate immune signaling pathway, have been used to explore the pathogenesis of various diseases, such as atherosclerosis, infection/sepsis ischemia–reperfusion, and autoimmune diseases (McCarthy et al. 2015; Hirata et al. 2013; Sorrentino et al. 2010; Chinnery et al. 2012; Meng et al. 2017). CpG ODN is also utilized for immunotherapeutic applications as immunoprotective agents, vaccine adjuvants, and anti-allergens (Kayraklioglu et al. 2021; Alberca-Custodio et al. 2020). Through TLR9-mediated innate and adaptive immune responses in immune cells, the host initiates a defense against invasive pathogens and eliminates harmful endogenous molecules (Kou and Wang 2023). While TLR9 activation by CpG ODN has shown therapeutic promise in cancer immunotherapy and antimicrobial defense, its functional outcomes exhibit striking context-dependency, influenced by factors including ODN class, target cell type, and disease milieu. For instance, recent work by Jiang et al. demonstrated that intraperitoneal administration of class A TLR9 agonists synergizes with anti-PD-1 therapy to enhance T-cell responses against colorectal peritoneal metastases, underscoring TLR9's potential as an immunoadjuvant in oncology (Jiang et al. 2022). Conversely, in models of bacterial peritonitis, TLR9 activation was shown to suppress the immunoregulatory functions of CD55^lo^ fibroblastic reticular cells (FRCs), impairing bacterial clearance and highlighting the dualistic roles of this pathway in stromal-immune crosstalk (Jiang et al. 2024). Studies also have shown that CpG ODN plays an immunomodulatory role in triggering the innate immune response to prevent infectious diseases (Wynn et al. 2009; Ut et al. 2016). However, the protective effect of CpG ODN targeting macrophages in APAP-induced liver injury has not been demonstrated.

In the present study, we found that CpG ODN-treated macrophages reinfusing to the animal reversed APAP-induced liver injury. In particular, transcriptome and metabolomes analysis revealed that pre-treatment with CpG ODN ameliorated mitochondrial damage and inhibited APAP-induced macrophage apoptosis through the immune regulating role of IRG1/itaconate metabolic pathway. According to the evidence shown in recent studies on the beneficial role of macrophages in APAP-induced liver injury, targeting macrophages may be a promising way to alleviate the resulting harmful effects (Roth et al. 2020; Li et al. 2022; You et al. 2013). Therefore, our study aimed to provide new therapeutic approaches for APAP overdose-induced liver injury and explore the potential mechanism to improve our understanding of macrophages in the context of liver homeostasis and diseases. This study may have long-term implications for better understanding the role of macrophages in the toxicology and pharmacology of APAP.

## Methods

### Animal model

Wild-type (WT) C57BL/6 mice aged 6–8 weeks (20 ~ 22 g) were purchased from the experimental animal center of Southern Medical University (Guangzhou, China). All mice were maintained in specific pathogen-free conditions and were housed in a temperature-controlled colony room on a 12/12 h light–dark cycle.

### Reagents

Acetaminophen, Percoll, collagenase IV and deoxyribonuclease I were obtained from Sigma-Aldrich (St. Louis, MO, USA). CCK-8 cell counting kit and Annexin V-FITC/PI apoptosis detection kit were purchased from Vazyme Biotechnology Company (Nanjing, China). CpG ODN 1826 was purchased from InvivoGen (San Diego, CA, USA). The tetrachloro-1,1’,3,3’-tetraethyl-imidacarbocyanine iodide (JC-1) kit and lactate dehydrogenase (LDH) cytotoxicity assay kit were purchased from Beyotime (Shanghai, China). Roswell Park Memorial Institute (RPMI) 1640, Dulbecco's modified eagle medium (DMEM), Dulbecco's phosphate-buffered saline (DPBS), and fetal bovine serum (FBS) were purchased from Gibco (Waltham, MA, USA). Antibodies for cleaved caspase-3, cleaved caspase-9, cleaved PARP, Bcl-2, BAX, phospho-NF-κB p65 (S536), Nrf2, GAPDH, β-actin, tubulin, Lamin A/C and anti-rabbit IgG, anti-mouse IgG, were all purchased from Cell Signaling Technology (Beverly, MA, USA). IRG1 antibody was purchased from Abcam (Cambridge, UK). JSH-23 was obtained from Selleck (Houston, Texas, USA). *Acod1* gene siRNA sequence: (1) sense: CCUGACAGAUGGUAUCAUUTT; antisense: AAUGAUACCAU-CUGUCAGGTT. (2) sense: CCAUAAAGUCACCCAAUAUTT; antisense: AUAUUGGGUGACUUUAUGGTT. (3) sense: CCGCGAGGCAUUGGCUAUUTT; anti-sense: AAUAGCCAAUGCCUCGCGGTT. (4) sense: GGGCCUCCAAGGAAACAAATT; antisense: UUUGUUUCCUUGGAGGCCCTT. *Acod1* promoter (2000bp) primer sequence: Forward: biotinylated-CATAAGTTACAAGTGTTACAGCCCT; Reverse: GGGCCTTTAGTGGACGTGT. Alanine aminotransferase (ALT) Assay Kit (C009-1–1) and Aspartate aminotransferase (AST) Assay Kit (C010-1–1) were purchased from Nanjing Jiancheng Bioengineering Institute (Nanjing, China). 4-Octyl itaconate and Hydroxychloroquine (HCQ) sulfate were purchased from MedChemExpress (Monmouth Junction, NJ, USA).

### Cell culture

RAW264.7 cells were obtained from the American Type Culture Collection (Manassas, VA) and were maintained in DMEM supplemented with 10% fetal bovine serum at 37 °C in a humidified atmosphere with 5% CO_2_.

### ALT/AST detection

Briefly, euthanize the mouse according to the authorized ethical guidelines. C57BL/6 mice were treated with APAP (300 mg/Kg) by intraperitoneal injection for 12 h after pre-treatment with CpG ODN (ODN 1826, 1.65 mg/Kg) for one hour in advance, then blood collection from cardiac puncture in mice was collected. After allowing the blood sample to stand at room temperature for 1 h, centrifuge for 10 min at 4 °C, then serum was obtained from supernatant. Serum ALT and AST was detected by detection kits using Reitman-Frankel colorimetric method according to the user manuals.

### BMDM isolation

Briefly, separate the femurs of mice under sterile conditions. Use tweezers and small scissors to cut off the muscles and fibrous tissue on the bone. Cut off both ends of the femur with a surgical knife or sharp scissors. Place the bone upright on top of a cell culture dish containing 5–7 mL of RPMI complete medium with forceps and carefully flush out the bone marrow with the syringe described above. Transfer all bone marrow from the cell culture dish to the cell sieve. Centrifuge cells at 600 × g for 4 min at 4 °C. Then cells were cultured in DMEM complete medium containing 40 ng/mL macrophage-colony stimulating factor (M-CSF), discarded half of the medium after 3 days and supplemented with fresh medium containing M-CSF. Furthermore, replaced the whole medium with fresh medium containing M-CSF on day 5, cells were cultured at 37 °C in a humidified atmosphere with 5% CO_2_ until day 7.

### Kupffer cells isolation

Kupffer cells were isolated according to the previous reference by Andreata, Francesco et al. (Andreata et al. 2021). Briefly, euthanize the mouse according to the authorized ethical guidelines. Firstly, perfuse the mouse liver with DPBS buffer containing 0.5 mmol EDTA until it swells and turns white. After cutting off the liver, placed the liver sample in a digestive solution containing 0.2 mg/mL collagenase IV and 5 unit/mL deoxyribonuclease I for dissociation. All liver cells obtained after dissociation were subjected to density gradient centrifugation using percoll and cell culture. Remove non-adherent cells, and the adherent cells obtained are Kupffer cells.

### Transwell co-culture experiment

In brief, 5 × 10^5^ Kupffer cells and AML12 cells were seeded in the upper and lower layers of the transwell chamber respectively, and different stimuli were given. DPBS was added to both layers of the ctrl group, while the upper layer of the 4-octyl itaconate group was pretreated with 4-octyl itaconate (250 μM) for 13 h. The lower layer of the APAP group was pretreated with APAP (5 mM) for 12 h, while the upper layer of the 4-octyl itaconate_APAP group was pretreated with 4-octyl itaconate (250 μM) for one hour in advance and the lower layer of APAP (5 mM) for 12 h. After the processing is completed, add a culture medium containing 10% CCK-8 solution in the lower layer by changing the used medium. After incubation for one hour, take the supernatant and measure the absorbance at 450 nm on SpectraMax M5 microplate reader.

### RNA sequencing

Briefly, RAW264.7 cells challenged with 5 mM APAP after pretreatment with and without 500 nM CpG ODN (ODN 1826) were cultured in 60 mm dishes. Then we extracted the total mRNA using trizol. MGISEQ-T7 platform was used for RNA sequence after the construction of the transcriptome library by Gene Denovo Biotechnology Co., Ltd (Guangzhou, China).

### Untargeted metabolomics

RAW264.7 cells challenged with 5 mM APAP after pretreatment with and without 500 nM CpG ODN (ODN 1826) were cultured in a 6-well plate overnight. Add 1 mL cold PBS buffer to each well of the cell culture plate and wash the cells 3 times. Cover the cells with liquid nitrogen to quickly stop cell activity. Add 400 μL cold methanol and gently swirl to ensure that all cells are covered by methanol. Then, store in a −80 °C refrigerator for at least 30 min. Add 100 μL pre-cooled ultrapure water and gently mix well, using a cell shovel to scrape the cells in methanol–water suspension. Wash the cells with 200 μL 80% cold methanol aqueous solution. Extract solution (acetonitrile: methanol = 1: 1, containing isotopically-labelled internal standard mixture) was added into the cell samples. Then, the ultra-high performance liquid chromatography q exactive mass spectrometry (UHPLC-QE-MS) analysis was used to detect after metabolites extraction. The quality control (QC) sample was prepared by mixing an equal aliquot of the supernatants from all of the samples.

### Target metabolome

In brief, 1 × 10^6^ RAW264.7 cells were seeded in a 6-well plate. Then, cell extract was obtained after cell lysis and solvent evaporation. The dried extract was recombined in 100 μL of methanol using an Agilent ZORBAX 300SB-C8 column (3.5 μm, 2.1 mm × 50 mm) at a column temperature of 25 ℃. The high-resolution accurate mass (HRAM) data was obtained using a liquid chromatography/quadrupole TOF mass spectrometer (Agilent 6530) in negative mode. The absolute concentration of itaconic acid was calculated based on the standard curve of itaconic acid.

### Cell viability and cytotoxicity assay

CCK-8 cell counting kit was used in the cell viability assay. Briefly, cell suspensions (100 μL/well) were seeded in 96-well plates at a density of 3 × 10^4^ cells/well for cell adherence. After overnight incubation, media were removed from each well, and plates were treated with APAP (1 mM, 5 mM, 10 mM) for 3 h, 6 h, 9 h, and 12 h. Ten microliters of CCK-8 solution were added to each well of the plate, and cells were incubated at 37 °C for four hours. Absorbances were read at 450 nm using a SpectraMax M5 microplate reader (Molecular Devices, Waltham, MA, USA).

Cell cytotoxicity was estimated using an LDH cytotoxicity assay kit followed by the user’s manual. Briefly, the cells were seeded onto 96-well plates at 3 × 10^4^ cells/well for cell adherence. After overnight culture, Opti-MEM media were removed from each well, and cells were treated with different concentrations of APAP (1 mM, 5 mM, 10 mM) for 3 h, 6 h, 9 h, and 12 h. Subsequently, the supernatant was incubated with 60 μL of mixed work solution for 30 min, and the absorbance of each well was measured at 490 nm using a SpectraMax M5 microplate reader (Molecular Devices, Waltham, MA, USA). The cytotoxic activity was calculated as: cytotoxic activity (%) = [(experimental LDH release − basic LDH leak)/(maximum LDH release − basic LDH leak)] × 100.

### Apoptosis assay

Apoptosis was detected by an Annexin V-FITC/PI apoptosis detection kit according to the manufacturer’s instructions. Briefly, cells (3 × 10^6^ cells/well) were seeded in 6 cm dishes and pretreated with or without CpG ODN (ODN 1826, 500 nM), and then subjected to different concentrations of APAP (1 mM, 5 mM, 10 mM) stimulation. Cells were collected after incubation with Annexin V-FITC/PI solution for 15 min at room temperature in the dark. Percentages of apoptotic cells were detected using a BD FACS flow cytometer.

### ROS detection

Dichlorodihydrofluorescein diacetate (DCFHDA) probe was used to detect the reactive oxygen species (ROS) production in APAP challenged RAW264.7 cells. Briefly, cells were incubated with 10 µM DCFHDA work solution for 30 min. Then wash 3 times using DPBS before incubating with DAPI 15 min. Collect the cells after DPBS washing 3 times. NIKON eclipse Ti2 fluorescence microscope was used to record the ROS production.

### Mitochondrial membrane potential assay

A mitochondrial membrane potential (MMP) assay kit with JC-1 was used to detect changes in MMP. Briefly, RAW264.7 cells were seeded in 6 cm dishes (3 × 10^6^ cells/well) for cell adherence overnight. After treatment with APAP (5 mM) or CpG (500 nM), cells were incubated with the JC-1 probe for 20 min in the cell incubator at 37 °C. Fluorescence intensity was monitored using the Amnis ImageStream Mark II Imaging Flow Cytometer (Luminex Corporation, Waltham, MA, USA). Decreases in red fluorescence and increases in green fluorescence indicated a decrease in MMP.

### Histopathological evaluation

Samples of liver tissues were fixed immediately with 10% neutral formalin and then embedded in paraffin. Tissues were cut into a thickness of 4–5 μm paraffin sections and pathological changes were evaluated by hematoxylin–eosin (HE) staining using light microscopy. Histological changes were evaluated by an ordinal scale for ranking the severity of hepatic injury following a protocol described previously (Ghaffari et al. 2011).

### DNA pull down

Magnetic bead preprocessing and nuclear protein pre-clearance: Transfer 50 µL of magnetic beads to a 1.5 mL EP tube and wash them three times with DPBS by resuspension and magnetic separation. After washing, resuspend the beads in 5 mg of nuclear protein sample. Incubate the mixture at 4 °C for 12 h with gentle rotation to allow preclearance. Following incubation, centrifuge the tube at 3,000 rpm for 5 min at 4 °C. Carefully transfer the supernatant to a new 1.5 mL EP tube using a pipette, avoiding disturbance of the bead pellet. Centrifuge the supernatant again at 14,000 rpm for 20 min at 4 °C to remove residual debris. Transfer the clarified supernatant to a fresh tube and store it on ice for subsequent DNA pull-down preparation. DNA-beads binding and protein incubation: In a separate tube, combine 50 µL of fresh magnetic beads with 10 µg of biotinylated *Acod1* promoter. Incubate the mixture at room temperature for 1 h with gentle agitation to promote DNA binding to the beads. Wash the DNA-bound beads three times with DPBS to remove unbound biotinylated DNA, using magnetic separation to pellet the beads between washes. Add the precleared nuclear protein supernatant to the DNA-beads complex. Incubate the mixture at 4 °C for 12 h with constant rotation to facilitate protein-DNA interaction. After incubation, wash the beads five times with ice-cold DPBS to eliminate unbound proteins and contaminants. Discard the supernatant completely after the final wash. Sample preparation and Western Blot detection: Resuspend the washed beads in 1 × SDS loading buffer. Heat the sample at 98 °C for 10 min in a thermal block to dissociate proteins from the beads. Briefly centrifuge the tube at 14,000 rpm for 1 min to pellet the beads. Transfer the supernatant to a new tube. Analyze the supernatant by Western Blot according to standard protocols, using appropriate primary and secondary antibodies to detect the target protein.

### Western Blot analysis

Briefly, RAW264.7 cells were seeded in 6 cm dishes (3 × 10^6^ cells/well) for cell adherence. After overnight incubation, the total protein was extracted by cell lysis buffer after treatment with APAP or CpG. The protein content in the samples was determined by a BCA protein assay kit (Thermo Fisher, Waltham, MA, USA) using a SpectraMax M5 microplate reader. Thirty micrograms of protein were subjected to SDS PAGE and transferred onto PVDF membranes. After blocking with 5% nonfat milk or BSA in Tris-buffered saline tween-20 (TBS-T) at room temperature for 1 h, the membranes were incubated with specific primary antibodies and secondary antibodies of goat anti-rabbit or goat anti-mouse at 4 °C overnight. Protein bands were detected by an Immobilon Western horseradish peroxidase (HRP) protein substrate (Merck Millipore, Billerica, CA, USA) and imaged with ChemiDoc™ Touch imaging system (BioRad, Hercules, CA, USA).

### Statistical Analysis

All data were analyzed using GraphPad Prism 7.0 (GraphPad Software Inc., San Diego, CA). The statistical analysis for multiple group experiments data was performed by one-way analysis of variance (ANOVA) followed by Tukey’s multiple comparisons test. A *P*-value < 0.05 was considered statistically significant for all experiments. All values are expressed as the mean ± standard deviation (SD).

## Results

### CpG ODN treated macrophages perform protective effect in APAP-induced liver injury in mice

**Fig. 1:**
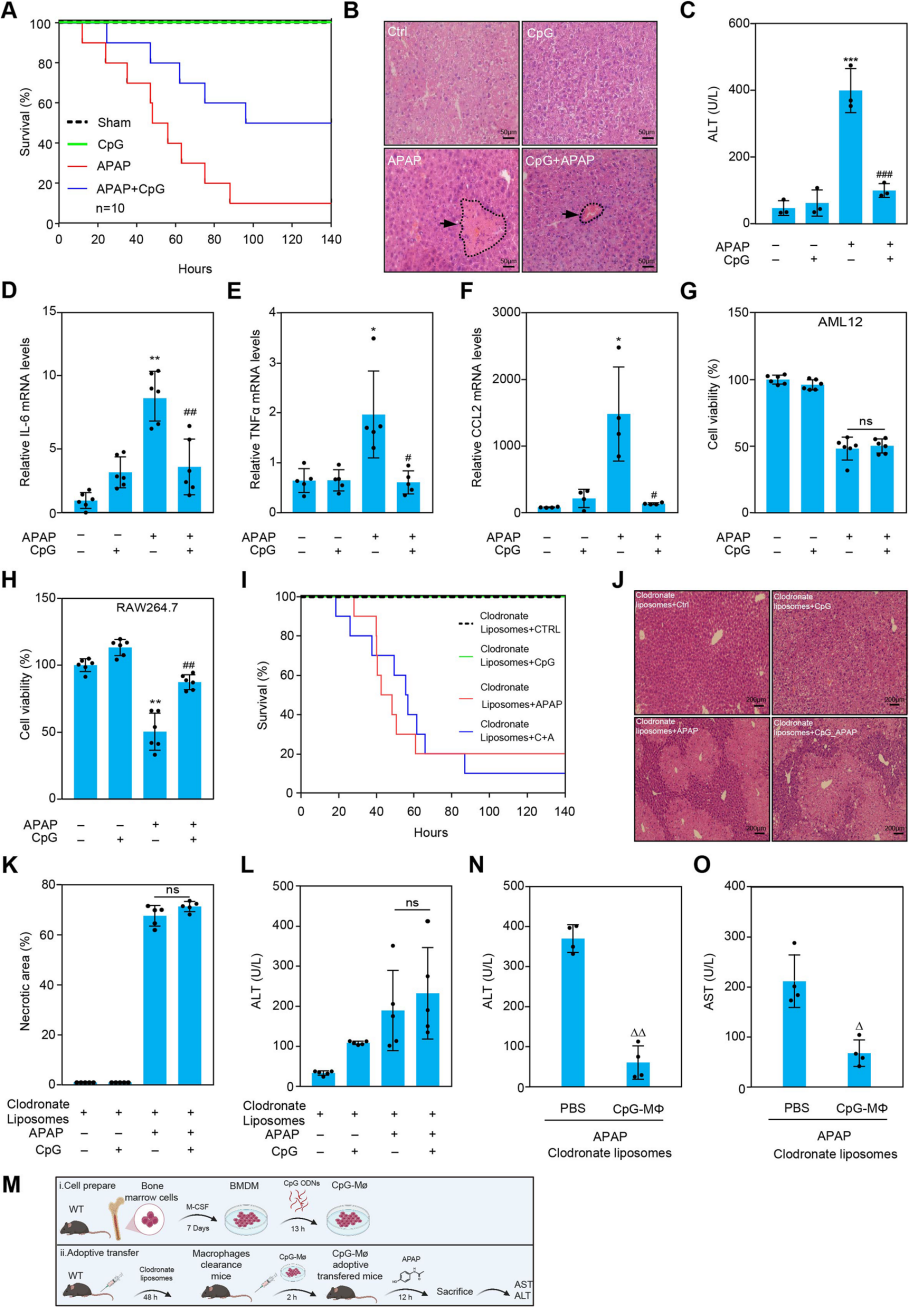
CpG ODN activated macrophages alleviated the APAP cytotoxicity in mice. C57BL/6 mice were treated with APAP (300 mg/Kg) by intraperitoneal injection, and then the survival rate of mice was observed (**A**). Hematoxylin–eosin (HE) staining (**B**) was used in histopathological evaluation of liver tissues by using light microscopy (× 400 magnification), scale bars, 50 µm. ALT assay was performed to evaluate the level of liver injury (**C**). Total mRNA was exacted from liver tissue. Then, the mRNA expression levels of *Il-6* (**D**), *Tnf-α* (**E**), and *Ccl2* (**F**) were assayed by qPCR. AML12 (**G**) and RAW 264.7 (**H**) cells treated by 500 nM CpG ODN (ODN 1826) or/and 5 mM APAP were used to determine the cell viability by CCK-8 assay. Animal survival rate was recorded to determine the role of macrophages in animal model after using clodronate liposomes (**I**). Liver tissue was used for histopathological evaluation by HE staining (**J**) and necrotic area analysis (**K**) (× 200, magnification), scale bars, 200 µm. ALT assay was used for liver function evaluation after clodronate liposomes, CpG ODN (ODN 1826, 1.65 mg/Kg) and APAP (5 mM) treatment (**L**). The adoptive transfer experiment was performed in vivo (**M**–**O**). The diagram of adoptive transfer experiment was shown (M). BMDMs treated by CpG ODN (ODN 1826, 500 nM) for 1 h were reinfused to animal model in vivo. Then, ALT (N) and AST (O) were detected 12 h later. Results are represented as means ± SD (*n* ≥ 3). * *P* < 0.05, **P < 0.01, ***P < 0.001, vs. control group; ^# ^*P* < 0.05, ^## ^*P* < 0.01, ^### ^*P* < 0.001, vs. APAP-treated group. ^Δ ^*P* < 0.05, ^ΔΔ ^*P* < 0.01, vs. PBS group

Acetaminophen causes severe liver damage and acute liver failure when overdosed. To ascertain the effect of CpG ODN (ODN 1826) on the survival of animal models with APAP-induced liver injury, the mortality of the mice challenged with APAP was observed after treatment with and without CpG ODN (ODN 1826) by tail vein injection for one hour in advance. Results showed that CpG ODN (ODN 1826) pretreatment remarkably increased the overall survival rate compared to APAP treatment alone (Fig. 1A). Histopathological evaluation and ALT measurements also revealed that the administration of CpG ODN (ODN 1826) significantly reduced APAP-induced liver injury (Fig. 1B and C). Meanwhile, the mRNA expression of inflammatory cytokines, including *Il-6*, *Tnf-α*, and C–C motif chemokine ligand 2 (*Ccl2*) in the liver remarkably decreased after CpG ODN (ODN 1826) treatment (Fig. 1D - F). The mRNA expression of genes related to hepatocyte protection *Il-10, Il-18bp, Il-6r* and the pro-inflammatory cytokine *Il-18* were also assayed. Results showed that pro-inflammatory cytokine *Il-18* mRNA expression increased in APAP- treated mice compared to that in APAP challenged mice with CpG ODN (ODN 1826) treatment. The mRNA expression of genes related to hepatocyte protection *Il-10, Il-18bp and Il-6r* was higher in the APAP mice with CpG ODN (ODN 1826) treatment rather than APAP treated mice (Figure S1a). It suggested that CpG ODN (ODN 1826) may reduce sterilized inflammation caused by APAP. Hepatocytes and non-parenchymal cells are the most important cells in the liver. Then we explored which type of hepatic cells can be protected by CpG ODN (ODN 1826) stimulated directly. Cell viability was measured respectively in AML12 hepatocytes and RAW264.7 macrophages challenged with APAP after CpG ODN (ODN 1826) treatment. It showed that there was no significant improvement of hepatocyte viability stimulated by APAP after CpG ODN (ODN 1826) treatment in AML12 than that in RAW264.7 cells (Fig. 1G and 1H). Cell viability was also determined in Kupffer cells challenged with APAP after CpG ODN (ODN 1826) treatment (Figure S1b). Results implied that hepatic parenchymal cells are not directly affected by CpG ODN (ODN 1826). Recent studies have shown that macrophages have a fundamental role in tissue repair and angiogenesis after APAP-induced liver injury. To determine whether CpG ODN (ODN 1826) exerts a protective effect by affecting macrophages directly. Firstly, we performed immunohistochemistry experiment to evaluated the distribution of macrophages stained with F4/80 antibody in vivo. Result showed that liver macrophages increased in CpG ODN (ODN 1826) treatment of APAP-treated mice than APAP-treated alone mice (Figure S1c). Then, we use a macrophage scavenger, clodronate liposomes, which can transfer chlorophosphate into cells and induce macrophage apoptosis, to evaluate the role of macrophages treated by CpG ODN (ODN 1826) in animal models. Immunohistochemical results showed F4/80^+^ macrophages in the liver were cleared completely after injecting clodronate liposomes for 48 h (Figure S1d). Then, the survival rate assessment data showed that there was no significant difference between only the APAP-stimulated group and APAP-stimulated with the CpG ODN (ODN 1826) pretreatment group (Fig. 1I). Histopathological evaluation and ALT measurements also suggested that there was no improvement after CpG ODN (ODN 1826) treatment in APAP-induced liver injury (Fig. 1J-L). Then, an adoptive transfer experiment in which CpG ODN (ODN 1826) -treated macrophages were infused into animal models via tail vein injection was performed to verify the protective role of CpG-treated macrophages (Fig. 1M). ALT and AST estimation implied that CpG-treated macrophage reinfusion can alleviate APAP-induced liver injury (Fig. 1N and O). All these results above suggested that CpG ODN (ODN 1826) performed a protective effect in the liver depending on the stimulation of macrophage directly.

### CpG ODN inhibits APAP-induced macrophage apoptosis through both extrinsic and intrinsic pathways

**Fig. 2:**
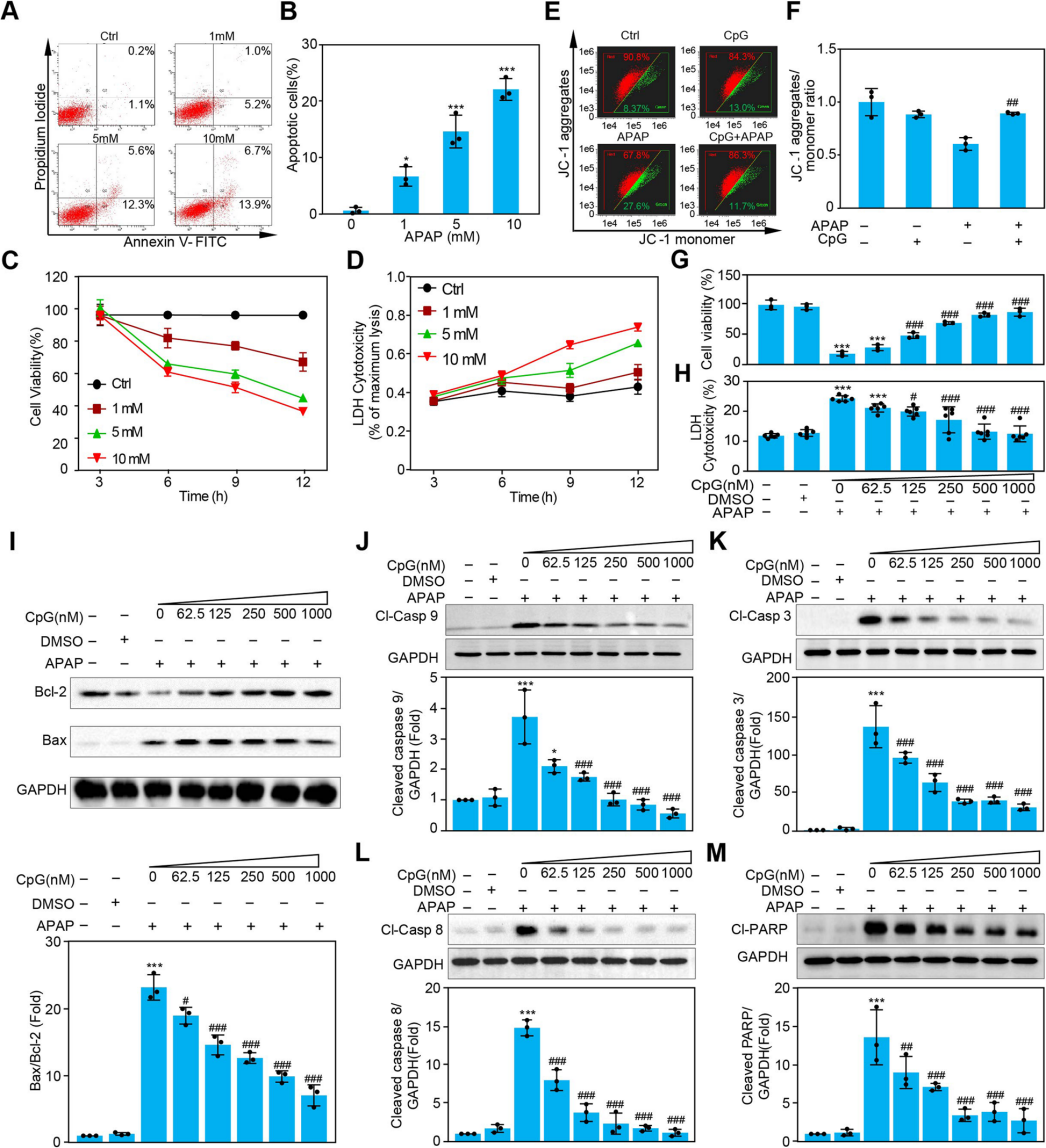
CpG ODN inhibits APAP-induced macrophage apoptosis. Flow cytometry analysis of Annexin-V and propidium iodide (PI) staining of apoptotic cells following APAP (1, 5, 10 mM) treatment for 12 h (**A**, **B**). RAW264.7 cells were exposed to APAP at various concentrations as indicated (1 mM, 5 mM, 10 mM) for 3 h, 6 h, 9 h, and 12 h. Then, cell viability and cytotoxicity were assayed by CCK-8 and LDH leakage detection respectively (**C**, **D**). Mitochondrial membrane potential (MMP) change in RAW264.7 cells with different treatments was detected by JC-1 probe using flow cytometry analysis (**E**, **F**). RAW264.7 cells were pretreated for one hour with different concentrations (nM) of CpG ODN (ODN 1826). Then, cell viability and LDH leakage were measured by LDH and CCK-8 assay after 12 h stimulation with APAP (**G**, **H**). After one hour of pretreatment with indicated doses of CpG ODN (ODN 1826), RAW264.7 cells were incubated with or without APAP (5 mM) for 12 h. Cell lysates were harvested and subjected to Western Blot analysis using antibodies targeted to apoptotic markers (**I**-M). Data were presented as means ± SD (*n* = 3). * *P* < 0.05, ** *P* < 0.01, *** *P* < 0.001, vs. control group (0 mM APAP or 0 h); ^# ^*P* < 0.05, ^## ^*P* < 0.01, ^### ^*P* < 0.001, vs. APAP-treated group

The cytotoxicity of APAP in hepatic cells has been well studied; however, the deleterious effect of APAP on immune cells has not yet been understood. Next, RAW264.7 cells were used to study the sterilized inflammation induced by APAP. Flow cytometer showed that apoptotic cells significantly increased in a dose-dependent manner after APAP exposure (Fig. 2A and B). A similar tendency that cell viability significantly decreased in a dose and time-dependent manner after APAP exposure was observed in the CCK-8 assay and LDH release detection (Fig. 2C and D). It has been suggested that APAP directly damages the macrophages. Moreover, the process is ongoing in an APAP dose-and time-dependent manner. Based on the protective effect of CpG ODN (ODN 1826) in APAP-challenged mice, we explored whether they also exhibited the same effect in vitro with different concentrations of CpG ODN pretreatment. As expected, the results of cell viability and the LDH release assay in RAW264.7 cells demonstrated that CpG ODN prevents APAP-induced cell death (Fig. 2G and H). Meanwhile, we also evaluate the macrophages of cell viability with different concentration of only CpG ODN stimulation, result showed that CpG ODN stimulation alone exhibited little effect on cell viability (Figure S2). Moreover, a change in mitochondrial membrane potential (MMP) is one of the symbol events in the early phase of cell apoptosis. The JC-1 assay was used to determine MMP changes in RAW264.7 cells. The results showed that the decrease in MMP caused by APAP was significantly reversed after pretreatment with CpG ODN (Fig. 2E and F). Thus, the effect of CpG ODN on the cytotoxicity of APAP may be associated with the inhibition of apoptosis in vitro. As we know, the cleavage of caspase is a critical step in the apoptotic cascade. To further investigate the influence of CpG ODN on APAP-induced cell apoptosis, caspase cleavage was detected. The results showed that the protein levels of cleaved caspases 9, 8, 3, and cleaved PARP decreased along with increasing concentrations of CpG ODN (Fig. 2J - M). We also observed a reduction in the level of the pro-apoptotic protein BAX and a concomitant increase in the anti-apoptotic protein Bcl-2. As a result, the ratio of BAX/Bcl-2 increased (Fig. 2I).

### RNA sequencing reveals IRG1 play a key role in CpG ODN treated macrophages in resistant to APAP cytotoxicity

**Fig. 3:**
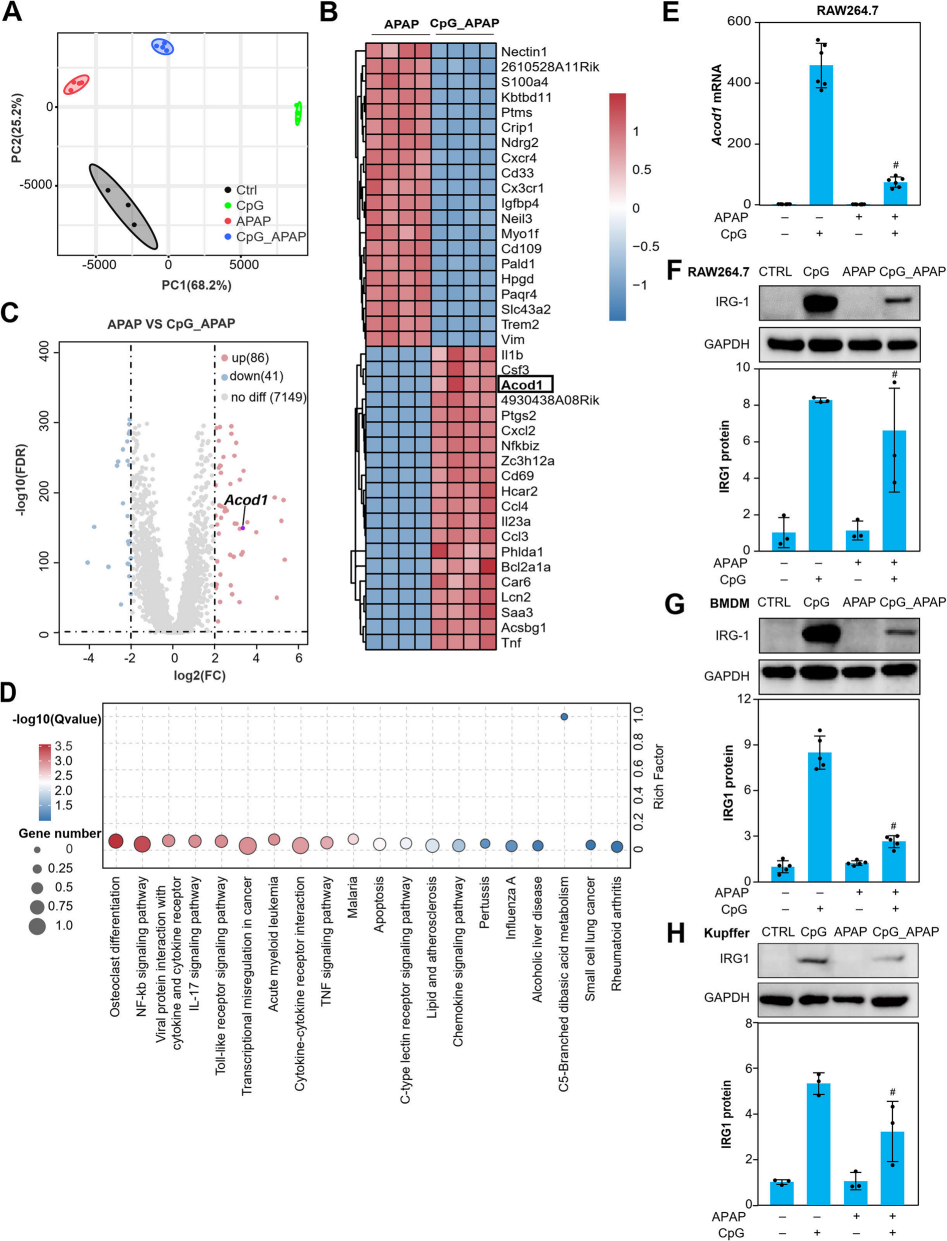
Transcriptome analysis reveals the key differential expression gene in resistant to APAP cytotoxicity. The total mRNA of RAW264.7 cells was exacted after different treatments as indicated. Then, library construction and sequencing were performed respectively. Based on gene expression information, principal component analysis (PCA) was conducted using R (‘gmodels’ package) (**A**). Hierarchical clustering of differential gene expression patterns and presentation of clustering results using heatmaps. We defined the differential expression genes (DEGs) with the parameter of false discovery rate (FDR) below 0.05 and fold change ≥ 2 using DESeq2 package. Then, we performed 126 DEGs between the APAP group and CpG_APAP group to cluster analyze genes based on the gene expression levels and presented the fold change of the top 20 up- and down- regulated genes by cluster analysis using heatmap. Red color, represents up-regulated genes; Blue color, represents down-regulated genes (**B**). Volcano plot analysis according to the FDR value and fold change were performed between APAP group and CpG_APAP group. Red dot, represent up-regulated genes; Blue dot, represent down-regulated genes (**C**). The 126 DEGs were used to perform pathway enrichment analysis comparing the whole genome background (**D**). The mRNA expression of *Acod1* gene was detected by qPCR in RAW264.7 cells (**E**). The protein expression of IRG1 was assayed by Western Blot in RAW264.7 cells (**F**), BMDM (**G**) and Kupffer cells (**H**). CpG_APAP group represents CpG ODN (ODN 1826) + APAP group. Data were presented as means ± SD (*n* ≥ 3). ^# ^*P* < 0.05, vs. APAP-treated group

To reveal the mechanism for the protective effect of CpG ODN (ODN 1826) in APAP-induced liver injury. RNA sequencing was performed in APAP stimulated RAW264.7 cells with and without CpG ODN (ODN 1826) treatment. Data derived from RNA sequencing were then presented by PCA analysis, which implicated that there were significant differences for pairwise comparison between groups in two dimensions of PC1 (68.2%) and PC2 (25.2%) (Fig. 3A). We screened the differential expression genes using criteria that log_2_ (FC) > 2, FDR < 0.05, and mean FPKM > 10 after normalizing the expression level by taking the log2 calculation value. In total, 126 differential expression genes were obtained between only the APAP stimulated group and the APAP stimulated with the CpG ODN (ODN 1826) treatment group. Among the 126 differential expression genes, we presented the fold change of the top 20 up- and down- regulated genes by cluster analysis which showed that *Acod1* was enrichment significantly in APAP stimulated with the CpG ODN (ODN 1826) treatment group over the APAP group (Fig. 3B). The top 20 upregulated genes also showed a close relationship using functional protein association networks (STRING database) analysis (Figure S3). Meanwhile, the significant differential gene *Acod1* was also determined by volcano plot analysis according to the *p* value and fold changes between the APAP group and the CpG_APAP group (Fig. 3C). The 126 differential expression genes were enriched by Kyoto Encyclopedia of Genes and Genomes (KEGG) pathway enrichment analysis that showed close association with alcoholic liver disease, apoptosis pathway, NF-κB signaling pathway, Toll like receptor pathway, and cytokine/chemokine signaling pathway (Fig. 3D). These results implied that the *Acod1* gene*,* which encodes the mitochondrial protein IRG1, may participate in the process of cell protection after CpG ODN (ODN 1826) treatment in APAP induced macrophage cell death model. Then the mRNA expression of *Acod1* and the protein IRG1 were both verified in RAW264.7 cells (Fig. 3E and F), results suggested that the expression of IRG1 protein was significantly increased after CpG ODN (ODN 1826) stimulated in RAW264.7 cells. The same tendency was observed in BMDMs (Fig. 3G) and Kupffer cells (Fig. 3H).

### Itaconic acid was identified as a key endogenous metabolite in the process of cell protection after CpG ODN stimulation

**Fig. 4:**
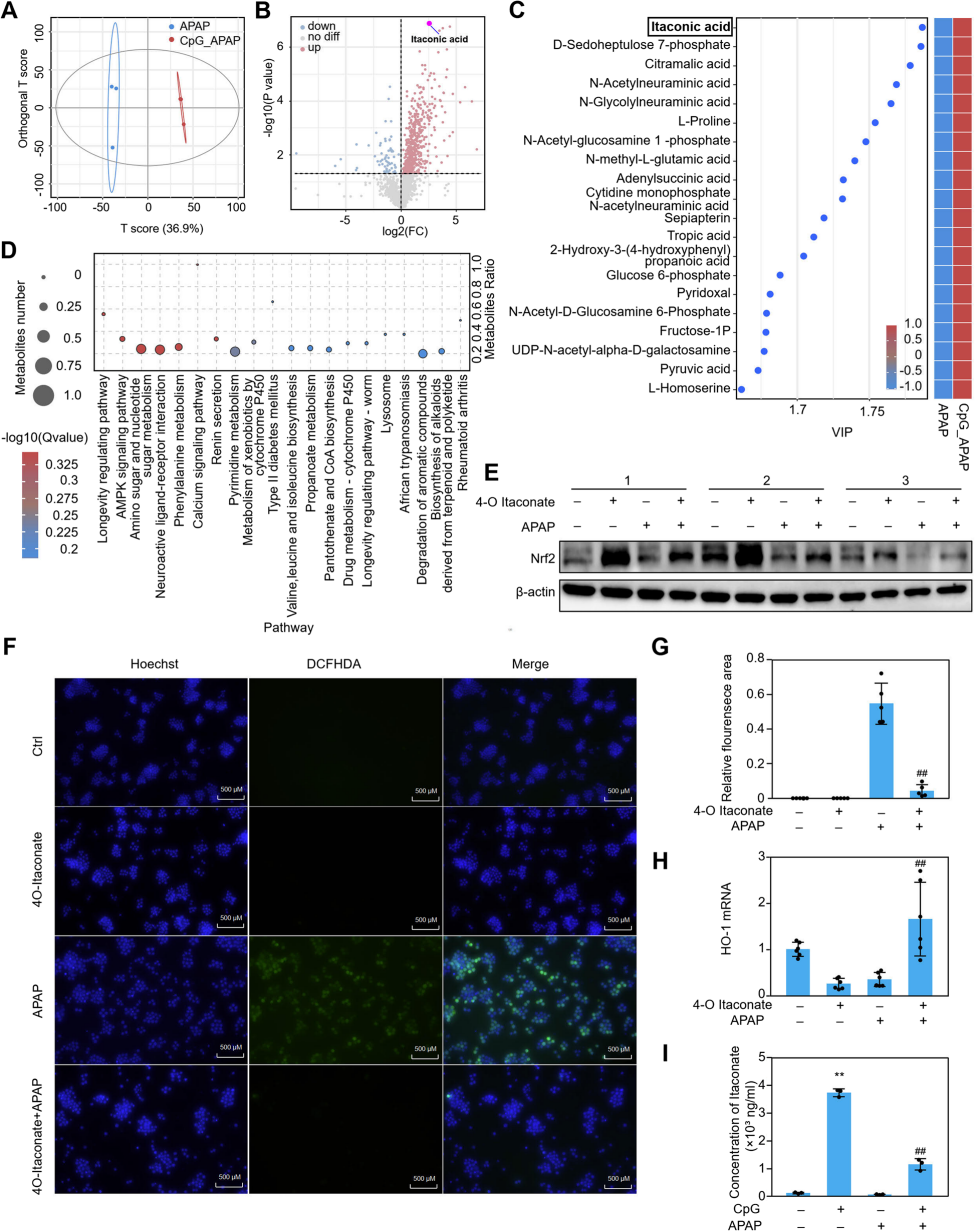
Untargeted and targeted metabolomics analysis and identification reveal the major differential metabolites in resistant to APAP cytotoxicity. RAW264.7 cells treated with different stimuli as indicated were lysed and extracted endogenous metabolites to perform untargeted metabolomics identification by UHPLC-QE-MS. According to the metabolite expression level, orthogonal partial least squares discriminant analysis (OPLS-DA) was used to construct a model between metabolite expression levels and grouping relationships to observe sample distribution and inter group difference between APAP group and CpG_APAP group using the R language (‘ropls’ package) (**A**). Volcano plot analysis according to the FDR value and fold change were performed between APAP group and CpG_APAP group. Red dot, represent up-regulated metabolites; Blue dot, represent down-regulated metabolites (**B**). According to OPLS-DA analysis, variable importance inprojection (VIP) values were obtained to visual the top 20 contribution of differential metabolites identified by LC–MS detection between APAP group and CpG_APAP group using ‘ggplot2’ package for R project (**C**). All the differential metabolites identified between APAP group and CpG_APAP group were performed KEGG pathway enrichment comparing the whole metabolites background by using ClusterProfiler package for R project (**D**). Nrf2 protein was assayed by Western Blot after 250 μM 4-O itaconate pretreatment for one hour with APAP(5 mM) treatment for 12 h. Three biological repeats were presented in the figure (**E**). After 250 μM 4-O itaconate stimulating for one hour, RAW264.7 cells were treated with 5 mM APAP for 12 h. Then, ROS production was detected by DCFHDA probe using fluorescence microscope. scale bar, 500 μm (× 200 magnification) (**F**). The bar chart is a statistical graph of fluorescence area for ROS detection (**G**). The expression of HO-1 mRNA in RAW264.7 cells was detected by qPCR with different stimulation (**H**). Target metabolome detection was used to determine the concentration of itaconate by UHPLC-QE-MS after 500 nM CpG ODN (ODN 1826) treatment (**I**). CpG_APAP group represents CpG ODN_APAP group. Data were presented as means ± SD (*n* ≥ 3). ** *P* < 0.01, vs. control group; ^## ^*P* < 0.01, vs. APAP-treated group. 4-O itaconate: 4-octyl itaconate

It is reported that IRG1 acts as an enzyme that catalyzes aconitate to produce itaconate in macrophages. Itaconate exhibits anti-inflammation and anti-oxidation functions in the biological process of cell metabolism. Untargeted metabolomics was used to explore the role of metabolite in macrophages for the protective effect of CpG ODN (ODN 1826). Liquid chromatograph mass spectrometer (LC–MS) data showed that there was a significant difference between the APAP group and the CpG_APAP group with a T score of 36.9% by orthogonal partial least squares discriminant analysis (OPLS-DA) (Fig. 4A). Then, according to the OPLS-DA analysis, variable importance inprojection (VIP) values were obtained to visualize the top 20 contribution of differential metabolites identified by LC–MS detection in these two groups (Fig. 4C). VIP map and heatmap show that itaconate was the highest abundance differential metabolite identified in the CpG_APAP group. The significant differential metabolite itaconic acid was also determined by volcano plot analysis according to the *p* value and fold changes between the APAP group and the CpG_APAP group (Fig. 4B). Then all the differential metabolites were mapped to the KEGG database to identify pathways that significantly enriched in differential metabolites. We present the top 20 enriched pathways using Clusterprofiler to visualize the result. The differential metabolites were observed close association in longevity regulating pathway obviously (Fig. 4D). These results implied that itaconic acid, as a metabolite catalyzed by IRG1, plays an important role in the process of cell protection for CpG ODN (ODN 1826) treatment. It is reported that itaconate exhibits an anti-inflammation role in sepsis, but the role of itaconate in APAP induced liver injury has not been elucidated yet. To explore further role of itaconate, we use 4-octyl itaconate, which is a cell-permeable itaconate derivative, to determine the relationship between itaconate and oxidative stress. The effect of 4-Octyl itaconate for penetrating into intracellular was validated by itaconate quantitated using UHPLC-QE-MS (Figure S4a). We found that the expression of Nrf2 protein was increased after 4-Octyl itaconate treatment in RAW264.7 cells (Fig. 4E). It suggests that itaconate may reduce APAP induced ROS production in macrophages. Then we evaluated the ROS abundance with and without 4-Octyl itaconate treatment in APAP challenged macrophages by using DCFHDA probe. The fluorescence intensity of ROS decreased significantly in APAP challenged cells with 4-Octyl itaconate treatment by using a fluorescence microscope (Fig. 4F and G). We also found that HO-1 mRNA expression was upregulated in APAP challenged macrophages with 4-Octyl itaconate treatment (Fig. 4H). Targeted metabolomics detection was used to verify that the concentration of itaconate was upregulated in CpG ODN (ODN 1826) treatment macrophages (Fig. 4I and S4b). All these results implied that the increased endogenous itaconate performed the antioxidative role in CpG ODN (ODN 1826) treatment macrophages. Furthermore, in order to determine the role of itaconate in liver macrophages in protecting against APAP cytotoxicity. We performed the co-culture experiments using Kupffer cells and liver parenchymal cells, AML12 cells. The results showed that treatment of macrophages with itaconate increased the viability of liver parenchymal cells challenged with APAP (Figure S4c). Overall, CpG ODN (ODN 1826) stimulated macrophages can alleviate APAP induced liver parenchymal cell damage by increasing the production of itaconate in macrophages.

### Activation of TLR9/NF-κB signaling pathway participate in the IRG1/itaconate mediated protective effect in overdose APAP induced liver injury

**Fig. 5:**
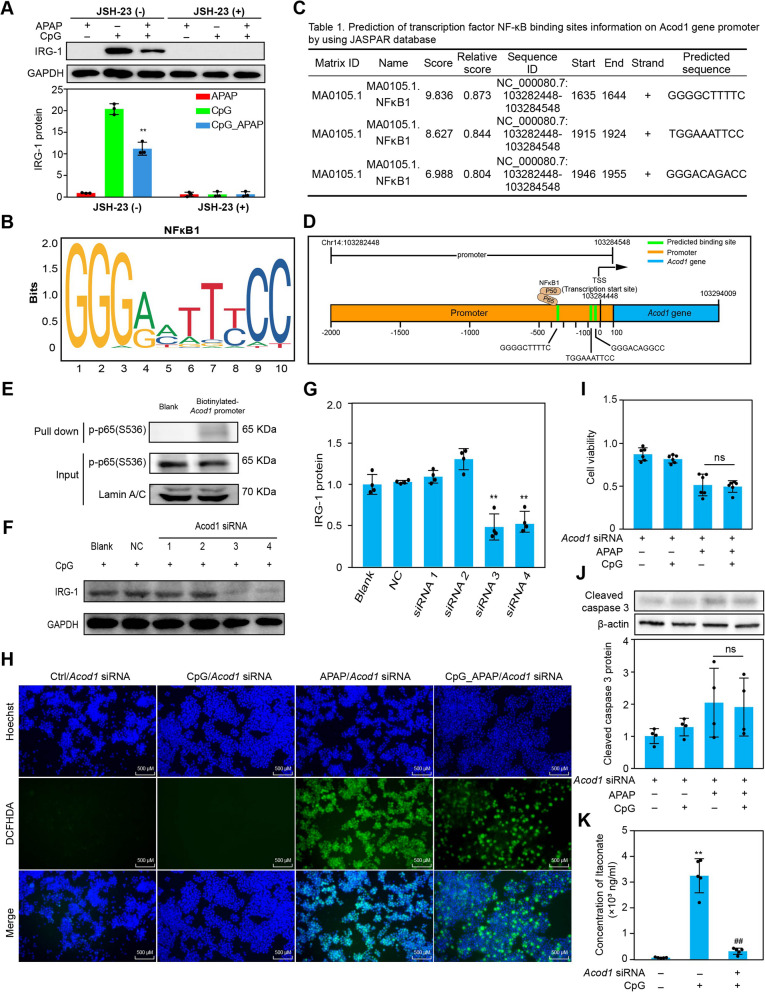
NF-κB plays as a transcript factor in mediating the IRG1 expression in CpG ODN activated macrophages. RAW264.7 cells were pretreated with 20 μM JSH-23 for 2 h before stimulation with 500 nM CpG ODN (ODN 1826) or/and 5 mM APAP, then, IRG1 protein was detected by Western Blot and statistic with bar chart (**A**). The probably binding sites motif of NF-κB was performed by JASPAR database (**B** and **C**). Structure diagram of *Acod1* gene on transcriptional regulation by NF-κB (**D**). Biotinylated *Acod1* promoter was obtained by PCR. Nuclear proteins were exacted from RAW264.7 macrophages challenged with 500 nM CpG ODN (ODN 1826) for 13 h. Phosphorylated NF-κB p65 was detected by Western Blot after nuclear protein pull down by the streptavidin beads binding with biotinylated *Acod1* promoter (**E**). Four *Acod1* siRNA probes were used to interfere the expression of IRG1 protein. RAW264.7 cells were treated with four *Acod1* siRNAs for 36 h respectively before 500 nM CpG ODN (ODN 1826) treatment, then, IRG1 protein was detection by Western Blot (**F** and **G**). After *Acod1* siRNA 3 treatment for 36 h, ROS production was detected by DCFHDA probe using fluorescence microscope with treatment as indicated. scale bar, 500 μm (× 200 magnification) (**H**). Then, cell viability was also detected by CCK-8 assay after *Acod1* siRNA 3 treatment for 36 h with or without CpG ODN (ODN 1826) and APAP stimulation (**I**). The cleaved caspase 3 was detected by Western Blot after *Acod1* siRNA 3 interference for 36 h with different treatment as indicated (**J**). The concentration of itaconate was quantitated by target metabolome detection using UHPLC-QE-MS after *Acod1* siRNA 3 treatment for 36 h with or without CpG ODN (ODN 1826) stimulation (K). Data were presented as means ± SD (*n* ≥ 3). ** *P* < 0.01, vs. control group; n.s., no significance. ^## ^*P* < 0.01, vs. CpG group

Based on the transcriptome evaluation, we found that IRG1 may perform a beneficial role in resistance to overdose APAP-induced liver injury. To further explore the underlying molecule function of IRG1 for the protection of CpG ODN (ODN 1826) in APAP-induced liver injury, we identified the metabolite itaconate, catalyzed by IRG1 through metabolomic analysis. According to the KEGG pathway enrichment of transcriptome analysis, toll like receptor signaling pathway and NF-κB signaling pathway may contribute to the process in the protective effect of IRG1/itaconate activated by CpG ODN (ODN 1826) in macrophages. To identify the potential molecular mechanisms underlying the transcription regulation of the *Acod1* gene on CpG ODN (ODN 1826)-treated macrophages, NF-κB inhibitor JSH-23 was used to observe the role of the NF-κB signaling pathway in vitro. Results showed that IRG1 protein was suppressed after NF-κB inhibited (Fig. 5A). Kupffer cells collected from C57BL/6 mice were also treated with JSH-23 to verify the role of the NF-κB signaling pathway in vivo and the same tendency was observed in Kupffer cells (Figure S5a)*.* Meanwhile, we determined the role of TLR9 signaling pathway in vivo by using TLR9 inhibitor, hydroxychloroquine (HCQ) sulfate. Result showed that the expression of IRG1 was inhibited by HCQ sulfate in liver tissue (Figure S5b). We further collected the Kupffer in vivo to detect the level of phosphorylated NF-κB p65 with different concentration of CpG ODN (ODN 1826) stimulation and evaluate the apoptosis related proteins in liver tissue (Figure S5c-e). These results were consistent with the tendency performed in vitro that CpG ODN (ODN 1826) treatment decreased the apoptosis in Kupffer cells and can activate the TLR9/NF-κB signaling pathway to regulate the mRNA expression of *Acod1* gene. It implied that NF-κB may bind to the *Acod1* gene promoter region to promote *Acod1* gene transcription. Then, we identified the potential binding sites of NF-κB which bind to the *Acod1* promoter region using JASPAR database (Fig. 5B-D). We also performed the experiment for DNA pull down to determined that phosphorylated NF-κB p65 bind to the *Acod1* gene promoter in regulating the transcription of IRG1. We used streptavidin beads and the biotinylated *Acod1* gene promoter to pull down nuclear protein, phosphorylated NF-κB p65 was detected by Western Blot. Results showed that phosphorylated NF-κB p65 was pulled down by biotinylated *Acod1* gene promoter (Fig. 5E). In addition, *Acod1* siRNA interference was used to knock down the expression of IRG1 in RAW264.7 cells to explore *Acod1* mediated the protective effect against APAP induced ROS accumulation (Fig. 5F-H). Results showed that CpG ODN (ODN 1826) treatment loses the protective effect in resistance to APAP induced ROS production after *Acod1* siRNA interference. The Same tendency was observed in cell viability detection and the activation of cleaved caspase 3 (Fig. 5I and J). Meanwhile, the concentration of itaconate was decreased after *Acod1* siRNA interference with CpG ODN (ODN 1826) stimulation (Fig. 5K). It represents that the production of intracellular itaconate was regulated by the expression of *Acod1.*

## Discussion

In the present study, we provide a new therapeutic strategy against APAP-induced liver injury. We found that CpG ODN (ODN 1826) exerts a protective effect on APAP-induced liver injury by the inhibition of macrophage apoptosis. The specific mechanism underlying the protective role of CpG ODN (ODN 1826) was due to the appropriate activation of the TLR9/NF-κB signaling pathway to upregulate the expression of IRG1 and the production of endogenous itaconate in macrophages. These findings provide a new light on the treatment for APAP-induced liver injury.

The mechanism of APAP-induced liver injury has been investigated and many biological processes were involved, such as APAP metabolism (Ghaffari et al. 2011; Lancaster et al. 2015; Saini et al. 2011), oxidative stress (Lee et al. 2015; Du et al. 2017), ER stress (Wang et al. 2016; Uzi et al. 2013), autophagy (Ni et al. 2012; Ni et al. 2016), sterile inflammation (McGill et al. 2012; Cai et al. 2014) and hepatic microcirculatory dysfunction (Ito et al. 2003; Ganey et al. 2007; Kopec et al. 2017). Most of these researches are focused on the damage of APAP to hepatocytes. Our study showed that targeting macrophages in APAP-induced liver injury could be an alternative approach to alleviate APAP induced liver injury. We provide evidence that CpG ODN (ODN 1826) exhibits the liver protective effect by its anti-apoptotic effect on macrophage in APAP induced liver injury. Furthermore, maintaining enough macrophages and activating the biological function of macrophages by CpG ODN (ODN 1826) are important for hepatocyte homeostasis in APAP-induced liver injury.

CpG ODN could be recognized by TLR9 to activate humoral and cellular immunity for preventing or treating cancer as one of the most effective immune stimulant adjuvants (Chen et al. 2021). The recognition of CpG ODN by TLR9 receptor recruited the intracellular Myd88, IRAK, TRAF6, activated MAP kinases and transcription factors such as NF-κB, AP-1, and IRF-7, subsequently regulating the expression of cytokine/chemokine genes, eliminating invading pathogens (Kumagai et al. 2008). Our results showed that the expression of the *Tnf-α*, *Il-6*, and *Ccl2* in the liver was significantly repressed by the administration of CpG ODN (ODN 1826) in APAP-challenged mice. This implied that the activation of the TLR9/NF-κB signaling pathway may play an uncommon role in resistance to APAP cytotoxicity in macrophages. In this study, conjoint analysis of transcriptome and metabolome revealed that IRG1/itaconate metabolic pathway participated in the process of the antiapoptotic and antioxidative effect of CpG ODN (ODN 1826) in macrophages of APAP-induced liver injury. It implied that CpG ODN (ODN 1826) initiated macrophage metabolic regulation is essential for resistant APAP-induced hepatocyte cytotoxicity. CpG ODN (ODN 1826) exhibited a strengthened immune regulation effect and enhanced innate immune response in APAP-induced liver injury through increasing endogenous itaconate production.

The role of macrophages in APAP-induced liver injury is controversial, as it has been demonstrated that these cells display pro-toxicant and hepato-protective functions. Here in this study, CpG ODN (ODN 1826) inhibits APAP-induced macrophage apoptosis to alleviate APAP-induced liver injury. One of the reasonable explanations was that macrophage exerted a proinflammatory role in the early stage of liver injury, resulting in the release of cytokines. In the late phase of liver injury, macrophage performs an anti-inflammatory function to engulf DAMP molecules and promote tissue repair. One previous study reported that CpG ODN induced early liver damage but provided a late window for protection against endotoxin-mediated hepatic injury (Slotta et al. 2006). This inspired us with the therapeutic potential of CpG ODN (ODN 1826) on APAP-induced liver injury.

There are also some issues in this study that need to be addressed in future studies. First, we strictly focused on the apoptosis of macrophages induced by APAP, excluding other important immune cells, such as neutrophils, which also showed the same tendency. Several studies have shown that CpG ODN inhibits neutrophil migration in vitro, leukocyte migration in vivo, and delayed apoptosis of neutrophil granulocytes (Admyre et al. 2015; Jozsef et al. 2004). Hepatic stellate cells (HSCs), the liver-resident fibroblasts with innate immune functions, serve as the principal cellular mediators of extracellular matrix (ECM) protein production during hepatic injury. In pathological conditions, HSCs undergo phenotypic transformation from a quiescent state to an activated fibroblast-like state, driving fibrogenesis. Notably, emerging evidence highlights the role of TLR9 signaling in modulating HSC activity. For instance, HSCs express functional TLR9, and its activation has been shown to exacerbate hepatic fibrosis by promoting pro-fibrotic responses (Gabele et al. 2008).

A study by Jiang et al*.* (2024) further elucidated TLR9's regulatory effects on fibroblastic reticular cells (FRCs), demonstrating that TLR9 activation suppresses proliferation, cytokine production, and retinoid metabolism in CD55^lo^ FRCs. Their work revealed that TLR9 deletion in CD55^lo^ FRCs enhances bacterial clearance and survival during peritoneal infection, suggesting context-dependent dual roles of TLR9 signaling in stromal cell regulation (Jiang et al. 2024). These findings raise important considerations regarding the potential limitations of CpG ODN-based therapies, particularly concerning unintended activation of profibrotic pathways in HSCs.

In our current model, comprehensive in vivo and cellular analyses demonstrate that the hepatoprotective benefits of CpG ODN-mediated primarily through macrophage modulation—outweigh localized adverse effects under the experimental conditions tested. However, we acknowledge that the broader implications of TLR9 activation in HSCs and other stromal populations remain to be fully characterized. Future studies will specifically investigate CpG ODN's direct and indirect effects on HSC activation states, cytokine profiles, and ECM remodeling dynamics in this injury model. This will clarify whether TLR9-driven macrophage-HSC crosstalk influences therapeutic outcomes or long-term fibrotic sequelae. Hence, further exploration of the cytoprotective mechanism of CpG ODN (ODN 1826) in other cell populations is required. Meanwhile, we only focused on the apoptosis influenced by CpG ODN (ODN 1826), other cell death types, such as autophagy, necroptosis, pyroptosis, entosis, ferroptosis, and parthanatos also need to be investigated in detail in future studies. It is well known that hepatic macrophages contain resident Kupffer cells (KCs) and monocyte-derived macrophages (MoMϕs). In our study, we did not discuss the role of these two subset macrophages activated by CpG ODN (ODN 1826) in the liver separately. We only used circulating monocytes derived macrophages to elucidate the mechanism of CpG ODN (ODN 1826) treated macrophages in resistant to APAP cytotoxicity rather than focus on the Kupffer cell isolated from the liver tissue. The reasons we considered this are: 1): both two subset macrophages (MoMϕs and KCs) in liver work together to maintain the liver homeostasis in clearing exogenous pathogens, cell debris and metabolic waste, tissue repair, and immune regulation. 2): Hepatic macrophages include MoMϕs and KCs origins are respectively from bone marrow and yolk sac-derived erythromyeloid progenitors (EMPs), but evidence also showed that yolk sac EMPs have the ability to develop into circulating macrophage precursors and to migrate to the liver (Gomez Perdiguero et al. 2015; Mass et al. 2016). In KC conditional depletion mice model, Monocyte macrophages can substitute the role of KC and present a KC phenotype. All these studies implied that MoMϕs, known to be the major contributor to the replenishment of the macrophage pool, are highly plastic which can differentiate into various phenotypes of hepatic macrophages in liver diseases (Sakai et al. 2019; Bonnardel et al. 2019; Wang and Kubes 2016). Thus, according to the plastic role of MoMϕs, we choose the bone marrow derived macrophages activated by CpG ODN (ODN 1826) to reinfuse back to the animal model and to clarify the underlying mechanism in hepaprotective effect. 3): Regardless of the two subset macrophages, it can be confirmed that the effect is mediated through the TLR9/NF-κB signaling pathway by CpG ODN (ODN 1826) administration. Last, we only used the JASPAR database to predict the binding sites of NF-κB, which act as transcription factor in participate in regulating the *Acod1* gene. More experiments should be performed to clarify the binding sites of NF-κB on *Acod1* gene promotor, like chromatin immunoprecipitation assay (ChIP). Moreover, the potential safety associated with the use of CpG ODN in vivo should be concerned. Clinical use of CpG ODNs raises safety concerns about autoimmune reactions (organ-specific/systemic) and toxic shock risk, as shown in animal models under specific conditions (Zeuner et al. 2003). While high doses or LPS co-administration caused toxicity in mice, normal-dose studies in animals and humans (Crohn’s, vaccines) showed no significant adverse effects (Klinman et al. 1997). CpG ODNs as immunoprotectants/adjuvants remain non-toxic in standard use, with ongoing clinical monitoring for reactions.

**Fig. 6:**
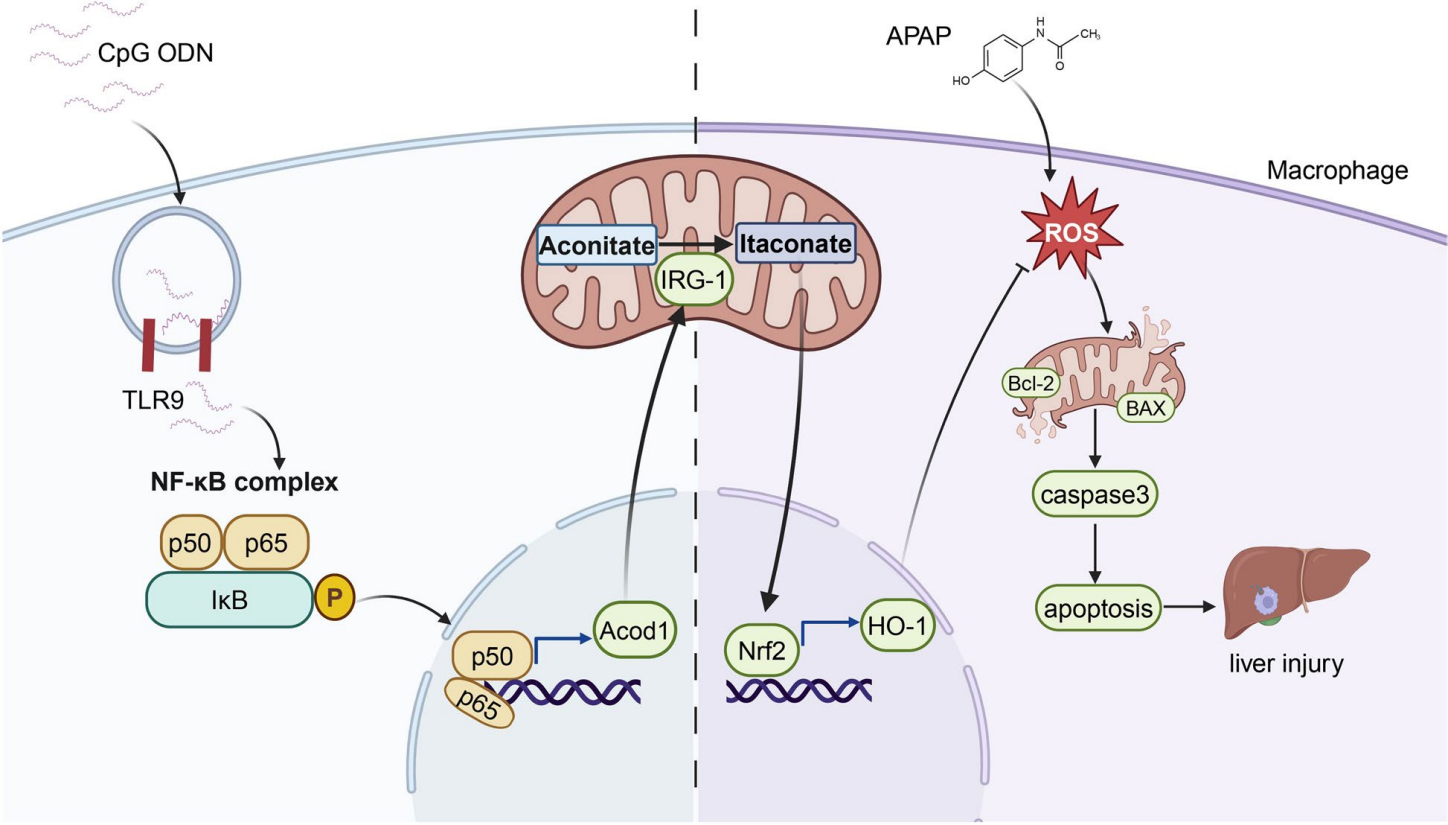
Schematic diagram of the mechanism in resistant to APAP induced liver injury by CpG ODN activated macrophages

In conclusion, our study found that CpG ODN (ODN 1826) treated macrophages play a protective role in APAP-induced liver injury. The mechanism underlying this phenomenon involved suppressing macrophage apoptosis after administration with CpG ODN (ODN 1826) by promoting the activation of the TLR9/IRG1/itaconate metabolic pathway. We believe that targeting macrophages could be an alternative approach for the treatment of APAP hepatotoxicity Fig. 6.

## Conclusions

CpG ODN (ODN 1826) alleviated liver injury induced by APAP through the activation of the TLR9/NF-κB signaling pathway in macrophages, upregulating of the expression of IRG1 protein, promoting the production of endogenous metabolite itaconate, and inhibiting macrophage apoptosis which was regulated by upregulating the expression of Nrf2 to inhibit ROS production.

## Supplementary Information

Supplementary Material 1.

Supplementary Material 2.

Supplementary Material 3.

Supplementary Material 4.

Supplementary Material 5.

## Data Availability

The original contributions presented in the study are included in the article materials; further inquiries can be directed to the corresponding authors.
