# Supplementary material for: CpG-oligodeoxynucleotides challenged macrophages ameliorate acetaminophen induced liver injury by activating TLR9/IRG1/itaconate metabolic pathway
Source: Mol Med. 2025 Aug 25;31:282. doi: 10.1186/s10020-025-01324-0 (PMC12379469; doi:10.1186/s10020-025-01324-0)
Supplement: Supplementary file 1 — Supplementary Material 1. [file 10020_2025_1324_MOESM1_ESM.pdf]

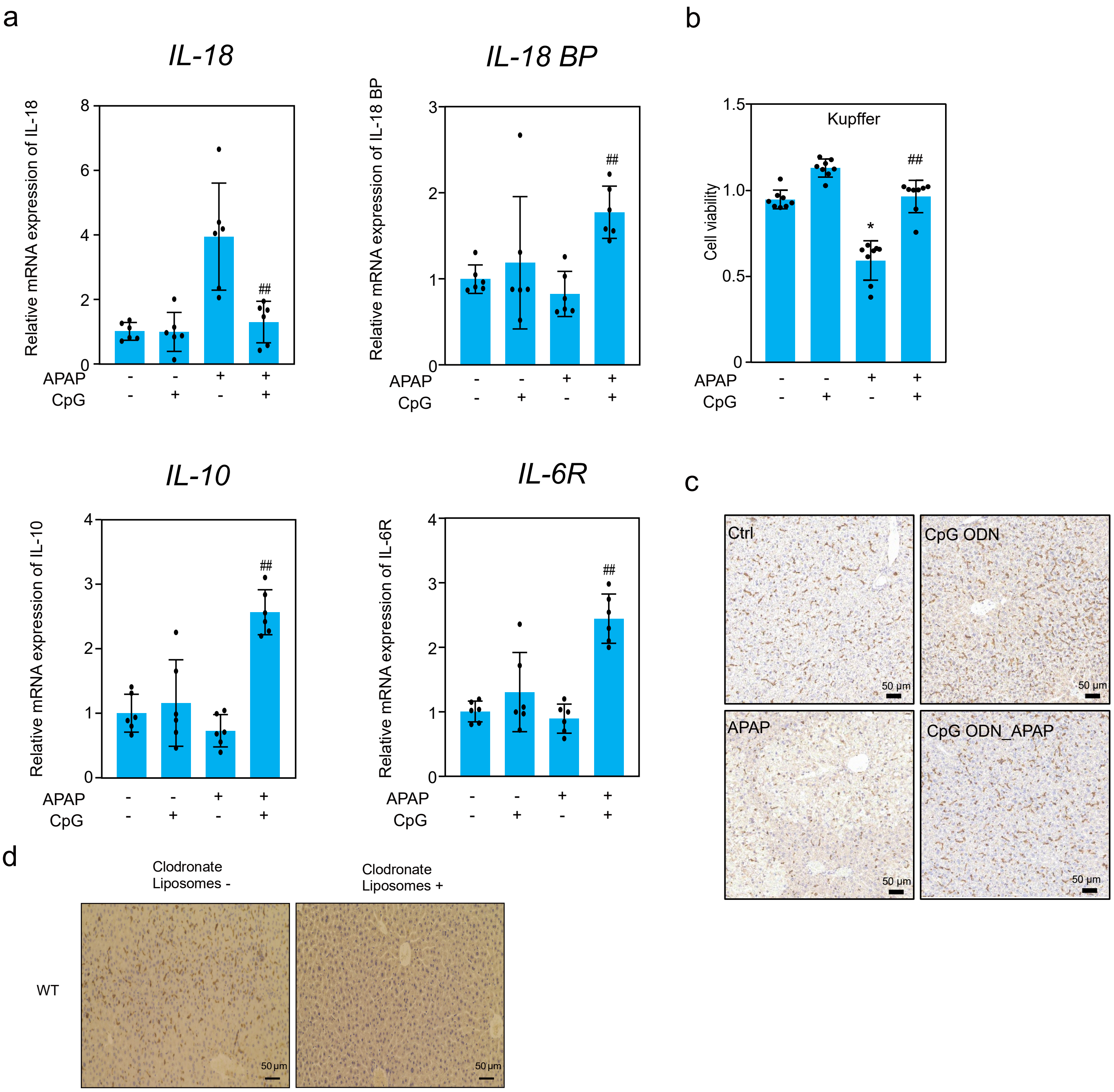

S1. C57BL/6 mice were treated with APAP (300 mg/Kg) by intraperitoneal injection for 12 hours after pre-treatment with CpG ODN for one hour in advance, then mouse liver was collected .
(a) The mRNA expression of IL-18, IL-18 binding protein, IL-10, IL-6R were detected by qPCR. ##, *P* < 0.01, vs. APAP group.
(b) Cell viability was determined by CCK8 assay in Kupffer cells after CpG ODN (500 nM) pretreatment for 1 hour in advance with APAP (5 mM) stimulation. Results are represented as means ± SD (n≥3). *, *P* < 0.05, vs. control group; ##, *P* < 0.01, vs. APAP group.
(c) mouse liver sample was collected and stained with F4/80 antibody for IHC evaluation by using light microscopy (×200 magnification). Scale bars represent 50 μm.
(d) The evaluation of macrophage clearance effect after tail vein injection of clodronate liposomes in wild type (WT) mice. Liver tissue were stained with F4/80 antibody with Immunohistochemistry assessment using microscope (×200 magnification), scale bar, 50 μm.

Figure S1
