# Supplementary material for: CpG-oligodeoxynucleotides challenged macrophages ameliorate acetaminophen induced liver injury by activating TLR9/IRG1/itaconate metabolic pathway
Source: Mol Med. 2025 Aug 25;31:282. doi: 10.1186/s10020-025-01324-0 (PMC12379469; doi:10.1186/s10020-025-01324-0)
Supplement: Supplementary file 2 — Supplementary Material 2. [file 10020_2025_1324_MOESM2_ESM.pdf]

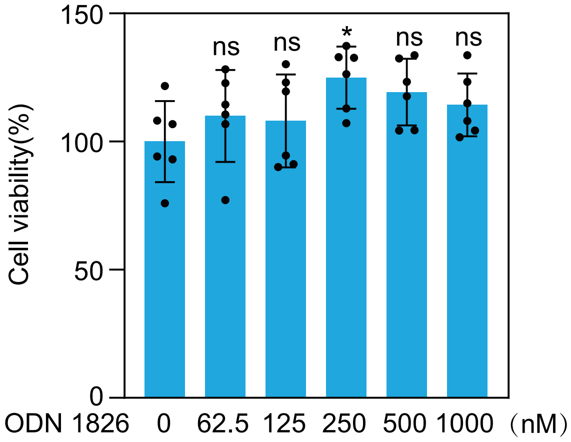

S2. The effect of CpG ODN on the cell viability of macrophages. **RAW 264.7 cells were challenged by different concentration of CpG ODN (0, 62.5, 125, 250, 500, 1000 nM) for 13 hours. CCK-8 assessment were used to determine the cell viability. ns, no significance, vs. ctrl group; *, *P* < 0.05, vs ctrl group.**

Figure S2
