# Supplementary material for: CpG-oligodeoxynucleotides challenged macrophages ameliorate acetaminophen induced liver injury by activating TLR9/IRG1/itaconate metabolic pathway
Source: Mol Med. 2025 Aug 25;31:282. doi: 10.1186/s10020-025-01324-0 (PMC12379469; doi:10.1186/s10020-025-01324-0)
Supplement: Supplementary file 3 — Supplementary Material 3. [file 10020_2025_1324_MOESM3_ESM.pdf]

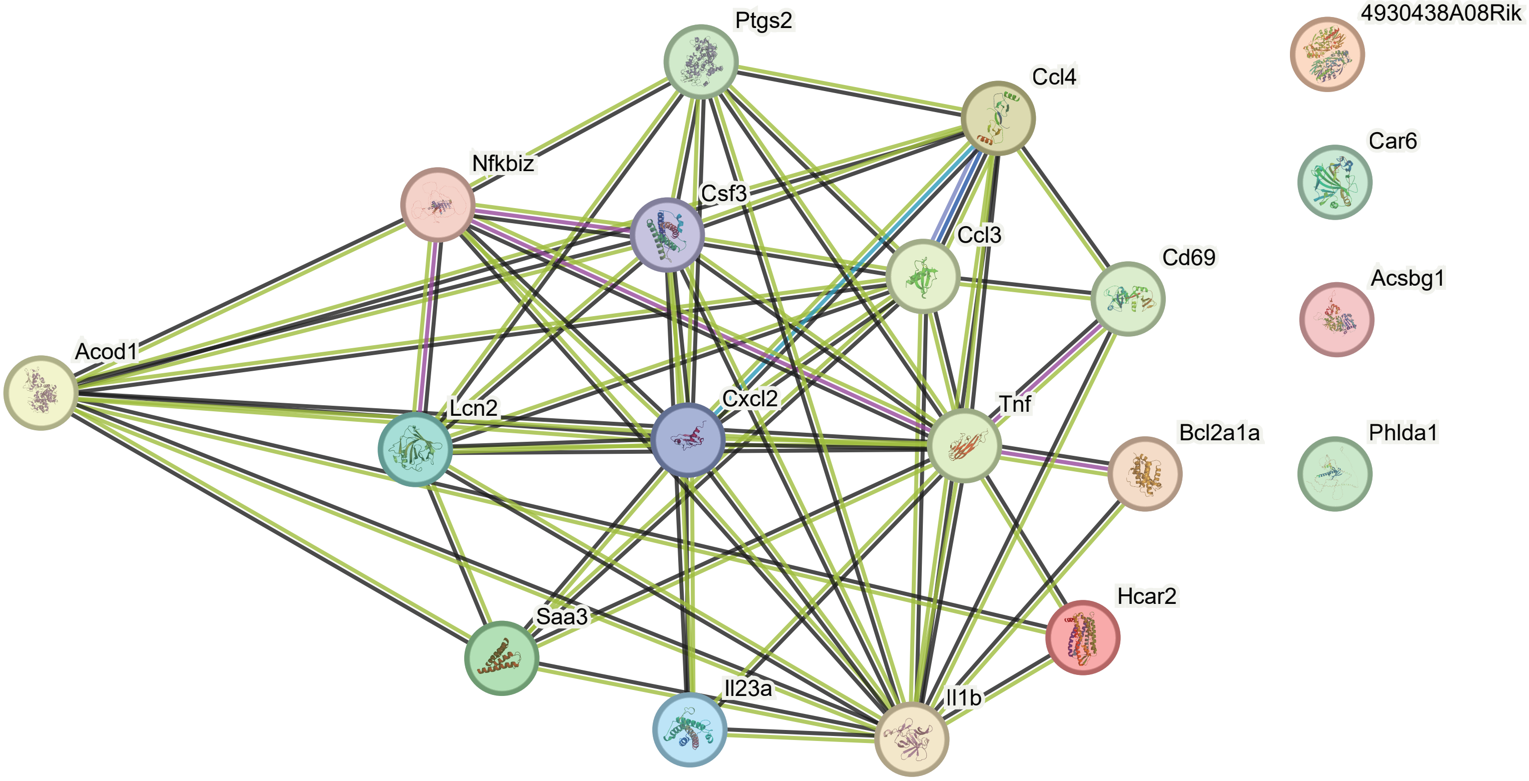

S3. Protein and protein interaction (PPI) network of top 20 up-regulated DEGs were performed by STRING database.

Figure S3
