# Supplementary material for: CpG-oligodeoxynucleotides challenged macrophages ameliorate acetaminophen induced liver injury by activating TLR9/IRG1/itaconate metabolic pathway
Source: Mol Med. 2025 Aug 25;31:282. doi: 10.1186/s10020-025-01324-0 (PMC12379469; doi:10.1186/s10020-025-01324-0)
Supplement: Supplementary file 4 — Supplementary Material 4. [file 10020_2025_1324_MOESM4_ESM.pdf]

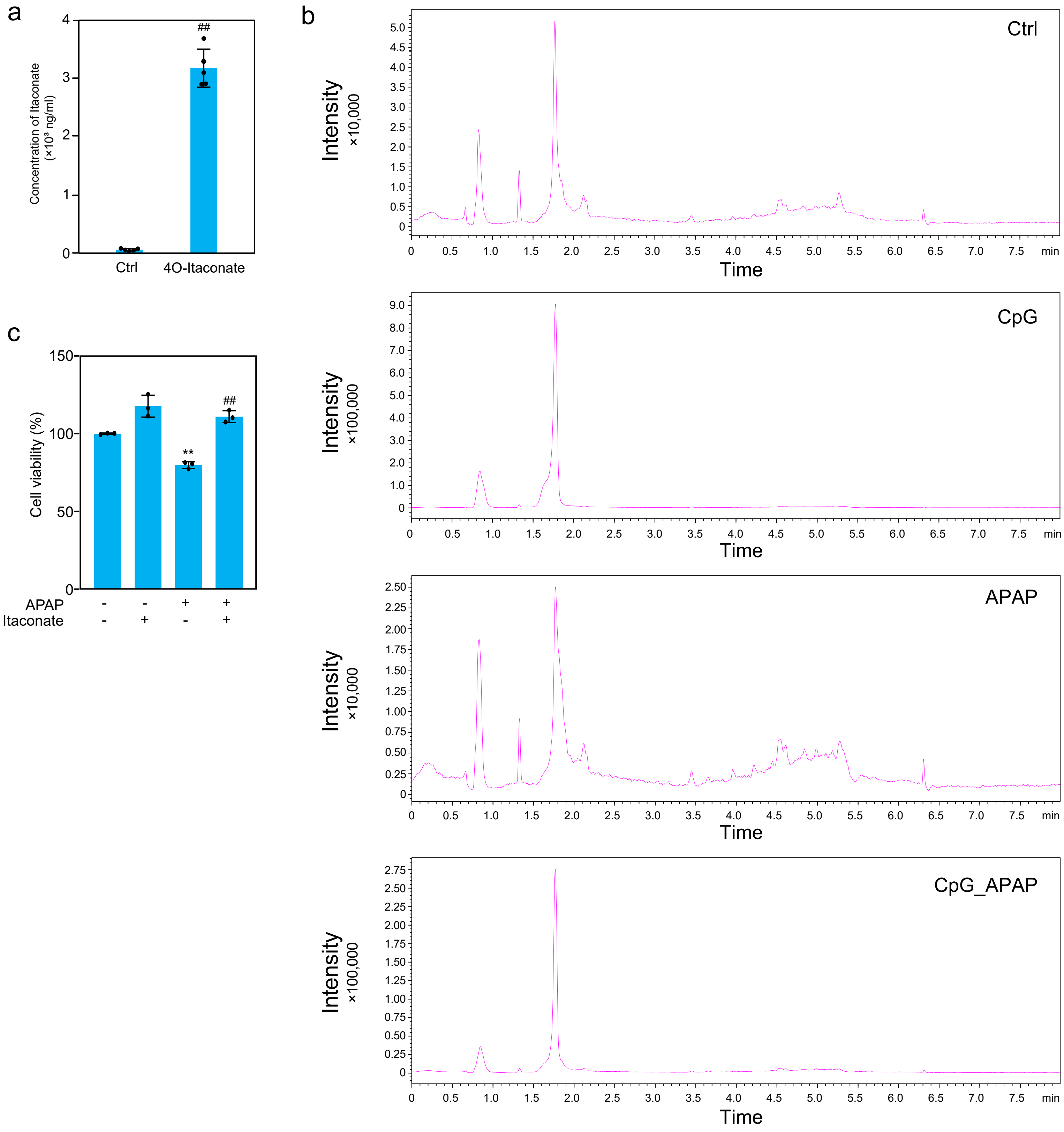

S4. (a) The concentration of intracellular itaconate was quantitated by HPLC after 4-Octyl itaconate stimulation. Data were presented as means ± SD (n≥3). ##, *P* < 0.01, vs. ctrl group.

(b) The chromatogram of itaconate which was identified by using HPLC for target metabolome.

(c) Kupffer cells and AML12 cells were co-cultured in transwell chamber. the upper layer of the Kupffer cells were pretreated with 4-octyl itaconate (250 μM) for 13 hours. The lower layer of the AML12 cells were treated with APAP (5 mM) for 12 hours. The cell viability of AML12 cells in the lower layer was detected by using CCK-8. Data were presented as means ± SD (n=3). **, *P* < 0.01, vs. ctrl group; ##, *P* < 0.01, vs. APAP group.

Figure S4
