# Supplementary material for: CpG-oligodeoxynucleotides challenged macrophages ameliorate acetaminophen induced liver injury by activating TLR9/IRG1/itaconate metabolic pathway
Source: Mol Med. 2025 Aug 25;31:282. doi: 10.1186/s10020-025-01324-0 (PMC12379469; doi:10.1186/s10020-025-01324-0)
Supplement: Supplementary file 5 — Supplementary Material 5. [file 10020_2025_1324_MOESM5_ESM.pdf]

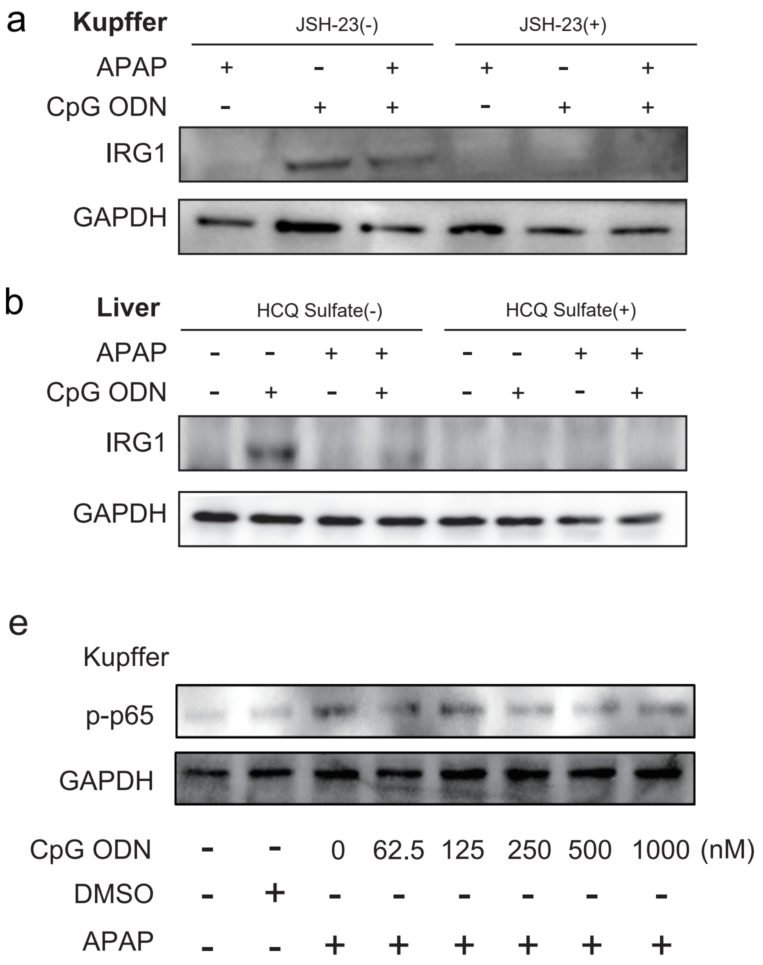

S5. Kupffer cells were isolated from WT C57BL/6 mice. After JSH-23 (30 μM) pre-treated for 2 hours, cells were then challenged with or without CpG ODN (500nM) for one hour in advance before APAP treatment. IRG1 protein were detected by western blot (a). C57BL/6 mice were treated with TLR9 inhibitor, hydroxychloroquine (HCQ) sulfate (50 mg/Kg), by intraperitoneal injection for 24 hours. Then liver tissue was collected from the mice which subjected to CpG ODN or/and APAP stimulation. Whole protein exacted from liver tissue were detected with IRG1 antibody by western blot (b). WT mice were challenged with APAP (300 mg/Kg) after pretreatment with CpG ODN 1826 for one hour in advance by tail vein injection. Then, liver sample and Kupffer cells were collected.Cleaved caspase 9 (c) and Bax protein (d) were detected by western blot in liver tissue. The p-p65 (e) protein were detected by western blot with APAP stimulation after different concentration of CpG ODN pretreatment for one hour.

Figure S5
